# Characterization of a Zika Virus Isolate from Colombia

**DOI:** 10.1371/journal.pntd.0005019

**Published:** 2016-09-21

**Authors:** Anismrita Lahon, Ravi P. Arya, Alexander R. Kneubehl, Megan B. Vogt, Natalie J. M. Dailey Garnes, Rebecca Rico-Hesse

**Affiliations:** 1 Department of Molecular Virology and Microbiology, Baylor College of Medicine, Houston, Texas, United States of America; 2 Integrative Molecular and Biological Science Program, Baylor College of Medicine, Houston, Texas, United States of America; 3 Departments of Medicine and Pediatrics, Sections of Infectious Diseases, Baylor College of Medicine, Houston, Texas, United States of America; Colorado State University, UNITED STATES

## Abstract

**Background:**

Zika virus (*Flavivirus* genus) is the first mosquito-borne virus known to cause high rates of microcephaly and abortion in humans. Typically, Zika virus causes a self-limiting, systemic illness; however, the current outbreak of Zika virus in the Americas has been associated with increased rates of fetal malformations and Guillain-Barré syndrome. Very few Zika virus isolates have been described in the literature, and live viruses are needed to perform studies of pathogenesis and to develop vaccines and treatments.

**Methodology/Clinical findings:**

We isolated Zika virus, strain FLR, directly from the serum of an individual infected in Barranquilla, Colombia (December, 2015). Here, we describe the patient’s clinical course and characterize strain FLR by its growth characteristics in mosquito and mammalian cells and its partial resistance to UV-inactivation. The full genome sequence of FLR was also analyzed (including the 3’ un-translated region), to determine its probable geographic origin, and to pinpoint structural differences from other Zika virus strains.

**Conclusions/Significance:**

We anticipate that the study of this low passage, clinical isolate of Zika virus, which is available for worldwide distribution, will help uncover the mechanisms of viral replication and host immune responses contributing to the varied and sometimes severe clinical presentations seen during the current epidemic in the Americas.

## Author Summary

Zika virus (ZIKV) is a mosquito-transmitted virus that causes a febrile illness characterized by a pronounced rash and occasional arthralgia. Serious complications from ZIKV infections were rarely reported until 2015, when Brazilian officials first reported an association between ZIKV infections and a dramatic increase in microcephaly and Guillian-Barré syndrome. Since then, ZIKV has been found in 39 countries in the Americas. Thus, the identification and characterization of ZIKV strains from the current outbreak are of utmost importance. In this study, we successfully isolated a ZIKV strain (ZIKV-FLR) from an individual who was bitten by mosquitoes while traveling in an area of Colombia that had ongoing dengue, chikungunya, and ZIKV transmission. Our analysis of the full genome sequence revealed that this virus strain may have originated in the Caribbean and was not directly imported from Brazil. We also discovered that this virus strain was partially resistant to inactivation by ultraviolet irradiation, when compared to dengue virus. Further work is needed to elucidate the underlying mechanisms behind this increased resistance and potential to cause more severe disease.

## Introduction

Zika virus (ZIKV) is a member of the *Flaviridae* family and *Flavivirus* genus and is primarily transmitted by *Aedes spp*. mosquitoes [1–3]. The current epidemic of ZIKV in the Americas has been the largest ZIKV epidemic to date; thirty-nine countries and territories in the Americas have reported ZIKV infections since its emergence into the western hemisphere in 2015 [4]. Zika virus infections were originally thought to be self-limiting with clinical manifestations of fever, headache, arthralgia, and maculopapular rash [1]; however, ZIKV infection has now been linked to the development of microcephaly, cerebral calcifications, and other fetal malformations in almost one third of infected, pregnant women studied in Rio de Janeiro, Brazil [5]. Retrospective studies of the 2013 outbreak in French Polynesia concluded that ZIKV-associated microcephaly may have occurred there as well [6].

Zika virus is not the only mosquito-borne virus known to cause microcephaly and fetal malformations. In domestic livestock (sheep cows, etc), infections with Wesselbrohn virus (*Flaviviridae*) or Rift Valley fever virus (*Bunyaviridae*) cause microcephaly and other fetal malformations at a rate similar to what is seen in the current ZIKV epidemic [7]. In humans, infections with Japanese encephalitis virus, West Nile virus, or dengue virus (all *Flaviviridae*) during pregnancy have been associated with central nervous system malformations in the developing fetus, but these occurrences are relatively rare [8]. Currently, ZIKV is the only mosquito-borne virus known to cause fetal malformations in humans at such a high frequency.

Mosquito control is the primary method available to prevent ZIKV infections; however, mosquito control has had little effect on reducing transmission of other viruses transmitted by *Aedes spp*. mosquitoes such as dengue and chikungunya [9]. Given the severity of the current ZIKV epidemic, work must be done to characterize and better understand the ZIKV strains circulating in the Americas. To that end, we isolated Zika strain FLR (ZIKV-FLR) from an individual infected in Colombia in December 2015.

## Methods

### Specimen collection and virus isolation

A blood specimen was collected from a 29-year-old nulliparous female who traveled to Barranquilla, Colombia. This sample collection protocol was approved by the Baylor College of Medicine Institutional Review Board (protocol H-38650); the adult participant provided written informed consent as approved by that board. The subject independently had her blood drawn and sent it to us for analysis. Serum was isolated from the blood sample and tested for dengue, chikungunya and ZIKV viral RNA by qRT-PCR. Serum was positive for ZIKV only and was used for C6/36 inoculation.

For virus isolation, one milliliter of the patient’s serum was used to infect C6/36 mosquito cells (*Ae*. *albopictus* larvae origin; ATCC) grown in Minimum Essential Medium (Corning) containing 2% fetal bovine serum, 1% glutamine and 1% penicillin and streptomycin. Presence of ZIKV was monitored via qRT-PCR in cell supernatant at 9 days post-inoculation. Virus (designated as ZIKV-FLR strain) was harvested and aliquots were stored in 30% FBS, at -70**°**C until use. Supernatant from ZIKV-FLR infected C6/36 cells was harvested periodically to check chronicity and stored at -70**°**C.

### Quantitative RT-PCR

Viral RNA was extracted from the supernatant of C6/36 cell culture infected with ZIKV and from dengue serotype 2 (DENV2) strain K0049 (SE Asian genotype, C6/36 passage 3) by using TRIzol LS reagent according to the manufacturer’s instructions (Thermo Fisher Scientific). Quantitative RT-PCR assays were based on the amplification of a ZIKV envelope gene region 3, and a DENV2 capsid region 1, using the ZIKV and DENV2-specific primers and probes, listed in Table 1. The reactions were performed using the TaqMan Fast Virus 1-Step Master Mix kit (Applied Biosystems), on a StepOnePlus Real-Time PCR system (Applied Biosystems). The concentrations of both ZIKV and DENV2 viral RNA (copies/mL) were estimated by using the standard curve generated from DENV2 transcripts. This standard curve was later compared to ZIKV RNA measured by spectrophotometry, to confirm their direct correlation.

**Table 1:** List of qRT-PCR primer/probes for ZIKV and DENV2 detection.

Virus	Primer/Probe	Genome Position	Sequence (5’-3’)	Reference
ZIKV	ZIKV 1086	1086–1102	CCGCTGCCCAACACAAG	[10]
	ZIKV 1107-FAM	1107–1137	AGCCTACCTTGACAAGCAGTCAGACACTCAA	
	ZIKV 1162c	1162–1139	CCACTAACGTTCTTTTGCAGACAT	
DENV	DENV2/141	141–160	GCTGAAACGCGAGAGAAACC	[11]
	DENV2/177-FAM	177–209	AGCATTCCAAGTGAGAATCTCTTTGTCAGCTGT	
	DENV2/234	212–234	CAGTTTTAITGGTCCTCGTCCCT	

### Full genome sequencing and analysis

Total RNA was isolated from supernatant of C6/36 cells infected with patient serum using TRIzol LS reagent (Thermo Fisher) following manufacturer’s protocol for 250μL of supernatant. Single-stranded cDNA was generated using SuperScript VILO (Thermo Fisher) on total RNA with extended incubation time (120 min at 42°C), followed by RNase free/DNase treatment (NEB), purification of the RNA (Qiagen RNeasy, RNA cleanup protocol) and subsequent second strand synthesis using NEBNext mRNA Second Strand Synthesis Module (NEB) following manufacturers’ protocols. Double-stranded cDNA was purified using NEB Monarch PCR and DNA Cleanup kit as recommended for fragment DNA. The sequencing library was prepared using the Nextera XT kit (Illumina) with purified double-stranded cDNA and then paired-end sequenced using Illumina’s MiSeq v2 kit (2 x 250bp) on a Miseq Benchtop Sequencer following manufacturer’s instructions (Illumina). Sequence reads were cleaned of Nextera adapters and Nextera PCR primers via Trimmomatic (v.0.35) and fastq (v.3.10.0) respectively. The reads were mapped to human genome (UCSC hg38) using BWA (v.7.12) to remove any potential human contaminating sequences. Reads that mapped to the reference genome and any unmapped reads paired with a mapped read were excluded using SAMtools (v.0.1.18). A dataset containing the *Ae*. *albopictus* genome (JXUM00000000.1) and all other *Aedes* sequences available at NCBI (19 Feb 2016) was created to remove contaminating reads. The reads previously cleaned of human contamination were mapped to the *Aedes* dataset via BWA (v.7.12) and the outcome was processed the same way it was for hg38. The remaining reads were further cleaned by removing any potential *Aedes* contamination based on the results of blastn (v.2.2.28+) against the *Aedes* dataset. Reads considered contamination were those that returned an e-value of ≤1.0E-5 to any of the aforementioned datasets via blastn. Quality control was performed after each removal using FastQC (v.1.6.0_34). The cleaned reads were assembled de novo using IVA (v1.0.3), which yielded the viral genome as a single contig [12]. Coverage was measured by mapping the reads used for the assembly back to the contig and the average depth of coverage was calculated using SAMtools (v.0.1.18). The assembled genome was submitted to GenBank via Sequin (v15.10) and was annotated using blastn (v.2.2.28+) and DNASTAR Lasergene (v.12) utilizing the polyprotein of a close relative, St. Louis Encephalitis virus (UniProtKB—P09732), and the protease cleavage sites as outlined previously [13], to delineate mature peptide regions. Potential N-glycosylation sites (Asn-Xaa-Ser/Thr) were predicted using NetNGlyc (v.1.0) for each protein of ZIKV-FLR, with the threshold for a positive prediction at 50% confidence.

To obtain the 5’ and 3’ un-translated ends of the viral genome, a 5’ and 3’ system for the Rapid Amplification of cDNA Ends (RACE) was used per the manufacturer's protocols (Thermo Fisher Scientific, USA). PCR amplicons were purified, sequenced bidirectionally using Sanger Sequencing, and the sequences assembled with the aid of DNASTAR Lasergene sequence analysis software (v.12). The 3’ UTR region of ZIKV-FLR was aligned with that of ZIKV-MR766 (KJ776791) and ZIKV-1225 (KU509998) using Clustal W software. The RNA secondary structures were predicted using the RNAfold server at ViennaRNA 19, and the structures shown here are the optimal energy folding patterns shown in the mountain format.

Evolutionary trees of genetic relationships of full genome nucleotide sequences from 10 previously reported ZIKV isolates (accession numbers EU545988 Micronesia-2007, KF268950 Central African Republic (CAF)-1976, KF268949 CAF-1980, KF268948 CAF-1976, KJ776791 Uganda-1947, KU365780 Brazil-2015, KU647676 Martinique-2015, KU681082 Phillippines-2012, KU720415 French Polynesia-2013, KU681081 Thailand-2014), and the strain FLR described here (KU820897 Colombia-2015), were generated by BEAST (v.2.3.2) [14]. Comparisons were limited to only those from ZIKV isolates and not sequences generated by combining amplicons [15]. Full genome nucleotide sequences of 11 ZIKV strains were aligned via ClustalW and were compared using the GTR substitution model and a relaxed molecular clock, with Bayesian MCMC algorithms. The phylogenetic tree shown in this study is the Maximum Clade Credibility tree generated by TreeAnnotator (v.2.3.2) (from 10 million) and visualized by FigTree (v.1.4.2).

### Immunofluorescent antibody assay

Vero cells (1x10^4^) were seeded onto chamber slides (Nunc, Lab-Tek II) 24 hours before infection. Cells were infected with ZIKV (using serial, ten-fold dilutions) for tissue culture infectious dose (TCID) determinations or ZIKV RNA copies (1x10^5^) for UV inactivation tests. At 5 and 10 days post infection of ZIKV or DENV2 respectively, cells were fixed in ice-cold acetone for 20 minutes, followed by incubation with anti-Flavivirus monoclonal antibody 4G2 (EMD Millipore) for 2 hours at 37°C. Cells were washed with PBS and incubated for 1 hour at 37°C with fluoresceinisothiocyanate (FITC)-conjugated anti-mouse IgG antibody (Life Technologies). Images were analyzed under a deconvolution microscope.

### Plaque assay

Vero cells were seeded at 3x10^5^ cells/well in a six well plate in DMEM (Gibco) containing 2% FBS (Atlanta Biologicals). Following an overnight incubation at 37°C with 5% CO_2_, cells were infected with ZIKV-FLR that was serially diluted (10^−2^ to 10^−6^) in DMEM with 2% FBS. Virus was removed after one hour, and cells were overlaid with 3ml of a 1:1 mix of 2% Seakem agarose (Lonza) and 2X DMEM with 10% FBS. After three days, cells were overlaid with 3 ml of a 1:1 mix of 2% Seakem agarose and 2x DMEM with 10% FBS and 0.03% neutral red [15]. Cells were monitored daily for plaque formation, until day 21.

### UV-inactivation of ZIKV and DENV

ZIKV and DENV were exposed to 1.2 or 0.6 joules/cm^2^ of UV irradiation with a Stratagene UV crosslinker [16]. Inactivation was tested by inoculating Vero cells and comparing irradiated and non-irradiated samples.

## Results and Discussion

The virus was isolated from the peripheral blood of a 29-year-old nulliparous female who traveled to Barranquilla, Colombia (IRB protocol H-38650). She first noted mosquito bites on her legs 2–3 days after arrival in Colombia. Two days after the first bites, she experienced fatigue for two days, followed by one day of ankle swelling, and then recovered. After returning to the US, nine days after first noting mosquito bites, she experienced onset of a pruritic rash on her palms, with progressive involvement over four days of her neck, trunk, and legs with minimal facial involvement. She was never febrile. On day two of the rash, the patient's leukocyte count was low (2,910 cells/mm^3^) with relative neutropenia (36%) and lymphocytosis (52%). Platelet count was normal (240,000 cells/mm^3^). Electrolytes and transaminases were within normal limits. On day three of the rash, she experienced 3–4 hours of swelling and stiffness of both hands and wrists; on day four, rash and fatigue began to resolve. She recovered fully by day six. Blood obtained on the second day of the rash was tested by qRT-PCR for dengue virus serotypes 2 and 3 [10], chikungunya virus [17], and Zika virus [11], as previously described. We did not detect dengue or chikungunya RNA; Zika virus RNA was detected, with an estimated concentration of 2,200 copies of viral RNA per milliliter. Of note, during the last week of December 2015, 6 laboratory-confirmed and 1200 clinically diagnosed cases of ZIKV infection were reported to the Colombian National Institute of Health from Barranquilla [18].

**Fig 1:**
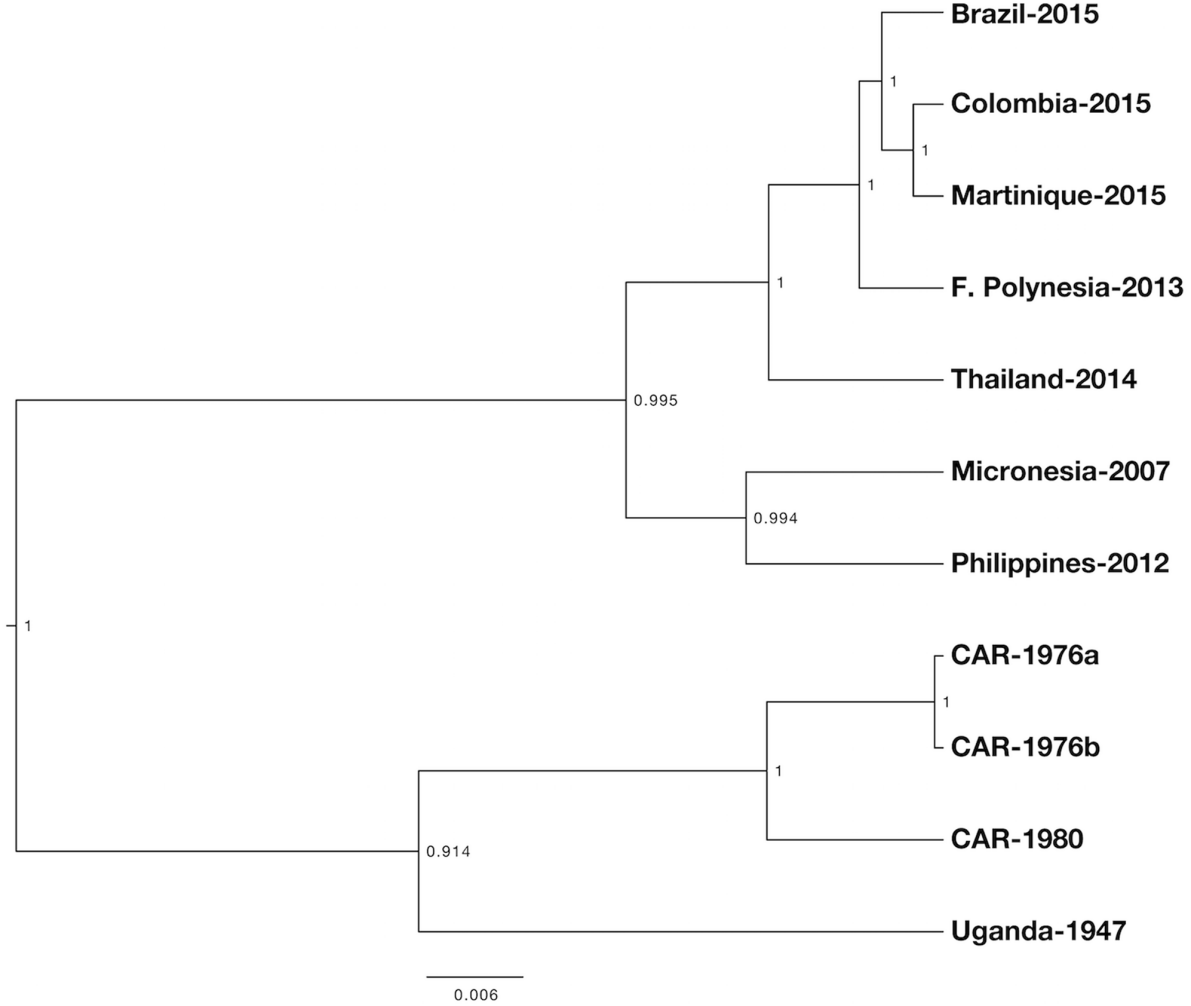
Phylogenetic tree of full genome nucleotide sequences of 11 ZIKV strains, using only those that were derived from viruses (i.e. not amplicons), available in GenBank. Alignments were done via Clustal W and analysis was done via Bayesian MCMC. Methods and accession numbers are provided in text. The scale represents substitutions per nucleotide site and the numbers on nodes are the posterior probabilities of branching (max = 1).

We infected C6/36 mosquito cells with ZIKV-positive patient serum and viral replication was monitored via qRT-PCR. The nucleotide sequences of ZIKV-FLR were determined by extracting RNA from the C6/36 cell culture supernatant (at day 14 post-infection) and performing Illumina deep sequencing. The genome (10,807 nucleotides in length) was annotated by comparison to another *Flavivirus*, and the N-glycosylation sites were predicted by NetNGlyc (Table 2). Phylogenetic trees were generated by Maximum Likelihood MCMC 10 analysis of 11 ZIKV strains, using the full genome sequences that had been aligned for virus isolates only (Fig 1). The resulting tree showed that ZIKV-FLR was most similar to those viruses from the recent Americas outbreaks, particularly to the strain from Martinique, 2015, which implies that the Colombian outbreak virus originated in the Caribbean and was not directly imported from Brazil. This conclusion was also reached recently by Lednicky *et al* when studying the geographic origin of a Haitian 2014 ZIKV [19]. They were able to isolate ZIKV in the Caribbean, at approximately the same time as it was introduced to Easter Island, off the Chilean coast, which suggests that ZIKV had been circulating in the Caribbean before the Brazilian epidemic (which began in 2015) [19].

**Table 2:** Predicted glycosylation sites for ZIKV-FLR.

Protein	Glycosylated	Amino Acid	Confidence (%)
Capsid	No	-	-
Pr	Yes	70	55.67
M	No	-	-
Env	Yes	154	63.08
NS1	No	-	-
NS2A	Yes	149	72.48
NS2B	No	-	-
NS3	Yes	158 |249|568	50.42|78.37|54.41
NS4A	No	-	-
Peptide 2K	No	-	-
NS4B	Yes	64|216	72.76|55.74
NS5	Yes	214	61.41

Using NetNGlyc (v1.0), the predicted N-glycosylation sites were determined for each protein of ZIKV-FLR. Amino acid corresponds to the residue number in each individual protein. “Confidence” is determined by NetNGlyc as the average prediction of all nine neural networks.

The study of the nucleotide sequences derived from ZIKV-FLR did not reveal any unusual genomic features or predict any protein structures that could explain increased virulence or different tropism phenotypes of the Americas outbreak when compared to the Asian and South Pacific outbreaks. Zika strain FLR has an N-glycosylation site at residue 154 of the E glycoprotein, which is conserved among Flaviviruses and putatively interacts with cellular receptors [20]. By including only sequences from viral isolates and not those obtained by enzymatic amplification and assembly of amplicons, we ensured that we were studying sequences from viruses capable of replicating.

**Fig 2:**
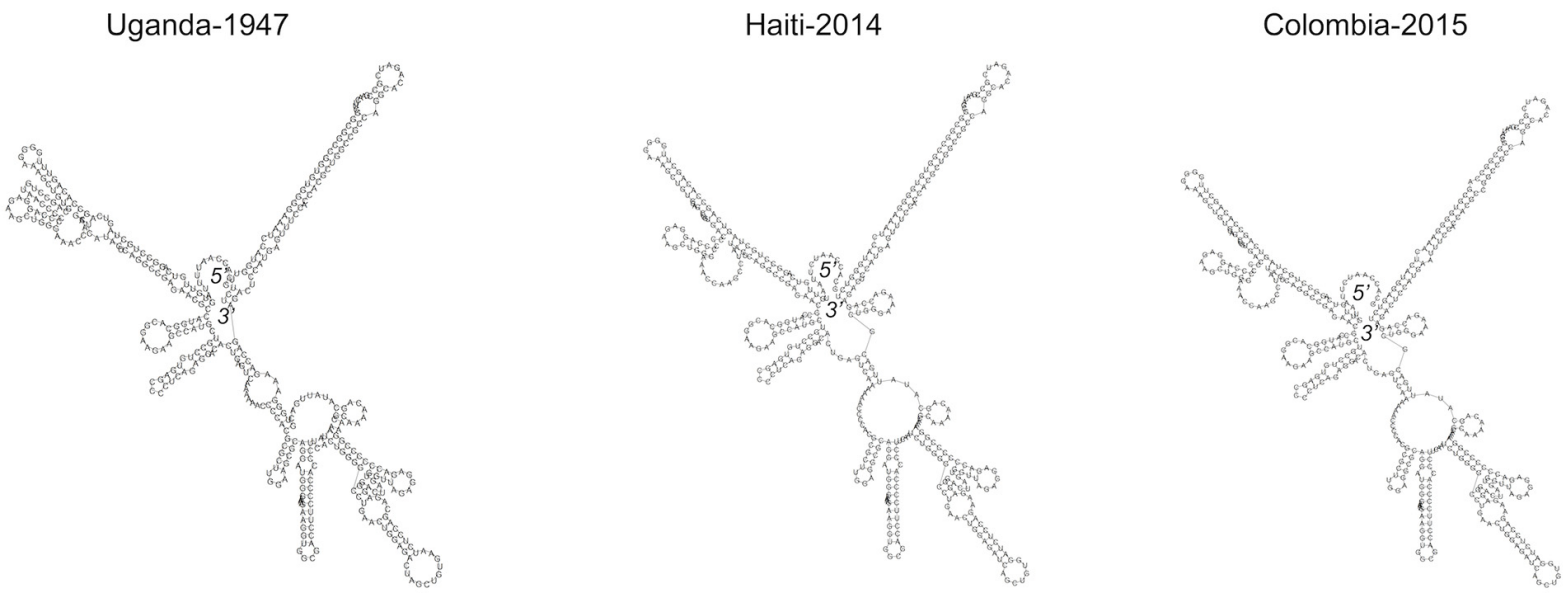
Predicted folding patterns for 3’ UTR regions of three ZIKV strains (428–429 nucleotides), with each labeled by country of origin and year of isolation. The 5’ and 3’ ends of the folded RNA are indicated. Non-coding nucleotides were aligned via Clustal W and the predicted RNA secondary structures were derived using RNAfold software.

We also determined the 3’ un-translated region (UTR) of ZIKV-FLR and compared it to two other ZIKV isolates, for which the 3’ nucleotides had been determined directly, via RACE [13, 19]. The RNA secondary structures of these noncoding regions are involved in determining the rates of *Flavivirus* RNA transcription and replication [21, 22]. The viral-encoded RNA Polymerase binds to this region, after the entire RNA genome circularizes, to form a replicative complex [22]. We compared the predicted folding patterns for the 3’ end of the three ZIKV for which sequences were available (nucleotide lengths were 428–429), using RNAfold software, available at the University of Vienna, Austria [23]. The older, Uganda-1947 strain formed a distinct folding pattern when compared to Haiti-2014 and Colombia-2015, with the latter two being almost identical (Fig 2). The extreme 3’ end of ZIKV-FLR had two variable nucleotides compared to Haiti-2014, and the so-called 3’ end stem loop was mostly conserved, differing by only one extra loop with no base-pairing (see top right loops in Fig 2). This region forms a very conserved 3’ stem loop pattern in all *Flaviviruses* studied to date, which may allow for high nucleotide plasticity [22]. These structures have been show to affect replication and virulence potential of DENV2 strains; however, it remains to be confirmed (via generation of recombinant ZIKV, mutagenesis, and chemical probing) whether these structures account for differences in replication and virulence among ZIKV strains [22, 24].

We observed chronic infection of ZIKV-FLR in C6/36 cells, with virus secreted into the cell culture supernatant more than 95 days post-infection, as tested via qRT-PCR. The concentration of viral RNA peaked at day 22 (4 x10^9^ copies/mL), but the number of infectious virions decreased (<4 x10^4^ TCID). Chronic infection of cell lines has been described previously for other Flaviviruses. These infections usually entail infecting mosquito cells with very high concentrations of virus, which induces the formation of defective, interfering particles (virus particles that do not contain complete viral genomes and are therefore not infectious) [25]. In this case, one full milliliter of serum was used to infect the cells because the serum had a very low concentration of viral RNA when quantified via qRT-PCR. Therefore, we did not expect this virus to produce a chronic infection, and it is unknown if establishing chronic infection is a consistent property of ZIKV. However, C6/36 cells, which are used routinely for arthropod-borne virus isolation, do not have a functional RNAi immune system [26], rendering these cells unable to detect and degrade intracellular viral RNA. This lack of RNAi may have contributed to the establishment of chronic infection.

**Fig 3:**
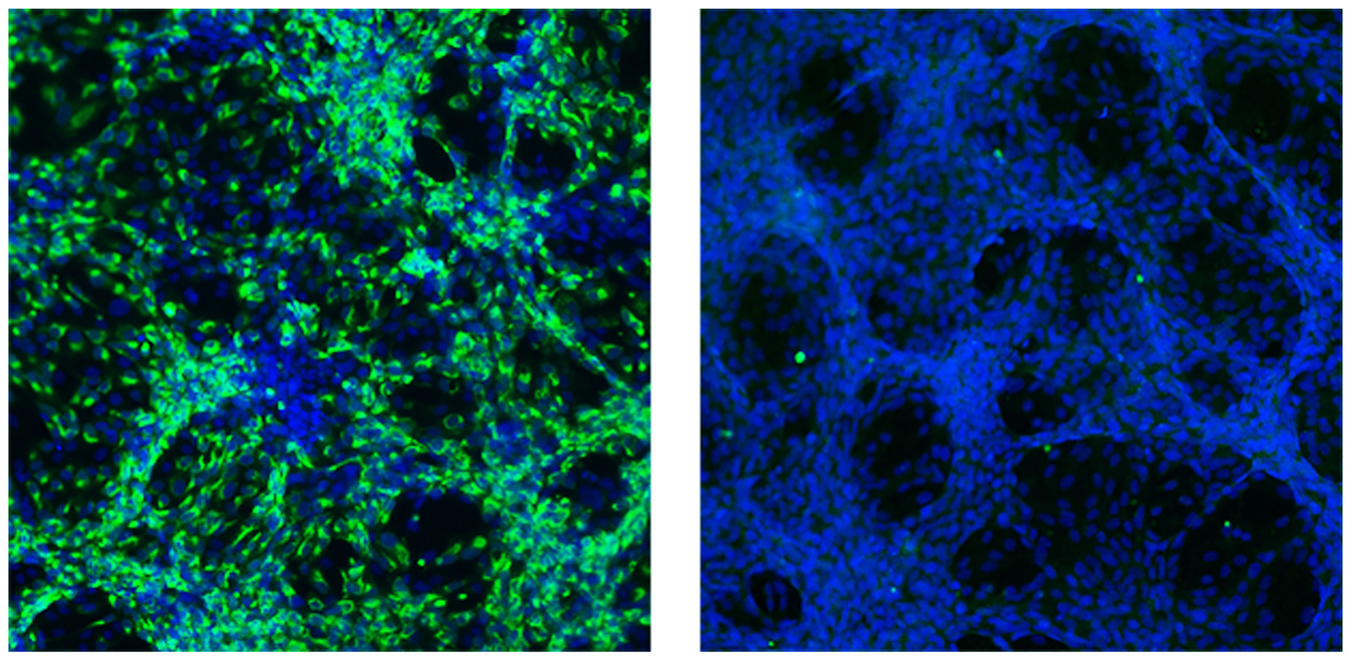
Limiting dilution immunofluorescence assay on Vero cells, to detect expression of ZIKV proteins, as a surrogate for replicating, infectious virus. The two panels represent the serial dilution at which a positive result was obtained (left), followed by the negative dilution (right). The highest positive dilution is expressed as TCID (Vero). 10X magnification.

We also infected Vero cells, which do not produce type I interferon, to determine the TCID or number of infectious particles in each sample. Cytopathic effects could be seen consistently on day 5 post-infection. We performed immunofluorescence tests for ZIKV envelope glycoprotein expression, using a monoclonal antibody (4G2, *Flavivirus*-specific) to confirm viral replication in these cells. The FLR strain also did not produce plaques in Vero cells (we attempted twice, with monitoring up to 21 days post-infection), as is common in other, low passage Flaviviruses (e.g., dengue viruses). This could be explained by a lack of adaptation to growth in Vero cells, at passage level 1 from patient serum; thus, plaque formation on monkey kidney cell line monolayers is probably not a ZIKV characteristic (others have used Vero passage 3+ virus; see ATCC). We compared ZIKV-FLR replication to a DENV2 virus strain that grows at very high rates in human target cells, but has not been shown to cause fetal malformations despite millions of human infections worldwide annually [24, 27]. Like ZIKV-FLR, DENV2-K0049 does not readily form plaques on Vero cells, but it does produce some cytopathic effects. Therefore, by inoculating Vero cells with limiting virus dilutions and testing for viral protein expression via immunofluorescence (Fig 3), we estimated that 1x10^4^ RNA copies equals 1 TCID in Vero cells for both low passage viruses. We consider the lack of plaque formation in Vero cells a good sign of the viruses not being adapted to grow in artificial systems, with no concomitant evolutionary selection for this *in vitro* phenotype. Thus, by using a low passage virus sample, we should better reflect natural viral diversity occurring in human plasma.

**Fig 4:**
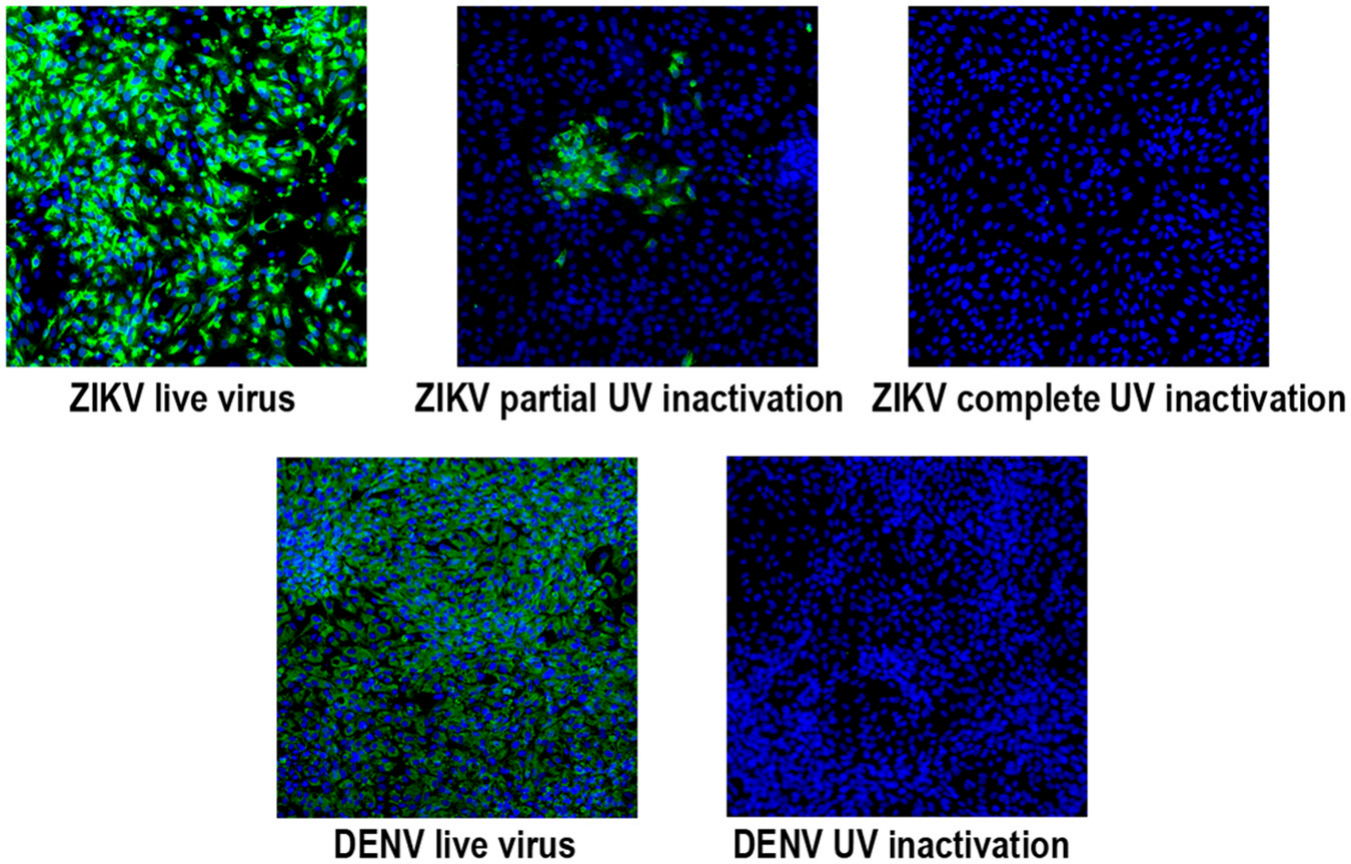
Immunofluorescence test on Vero cells, to detect inactivation of ZIKV-FLR and DENV2-K0049 infectious virus particles by UV-illumination. ZIKV -FLR was treated twice, for a total of 1.2 joules/cm^2^, for inactivation, while DENV2 needed only 0.6 joules/cm^2^ for inactivation.

We performed ultraviolet (UV) light inactivation of both ZIKV-FLR and DENV2-K0049 to differentiate between RNA replication versus RNA genome stability (or breakdown, in recipient cells). Zika virus and DENV2 were exposed to 0.6 or 1.2 joules/cm^2^ of UV illumination, in a Stratagene UV crosslinker, and used to infect Vero cells. The number of infectious particles was estimated by endpoint dilution experiments with immunofluorescence, as before. DENV2 was inactivated by one exposure to 0.6 joules/cm^2^ of UV, while ZIKV was not inactivated; the UV dose had to be doubled to eliminate ZIKV-FLR replication in Vero cells (Fig 4). Combined with resistance to heat inactivation described for the French Polynesia strain H/FP/2013, this resistance to UV irradiation suggests that there are structural factors (genomic and proteins) that increase ZIKV stability [28]. Whether this resistance has an effect on ZIKV tropism or pathogenesis in humans, and/or transmission by mosquitoes remains to be determined.

### Accession code and virus isolate

The ZIKV-FLR sequences are available in GenBank, accession number KU820897. The virus isolate is available through BEI Resources and ATCC.
